# Want More? Learn Less: Motivation Affects Adolescents Learning from Negative Feedback

**DOI:** 10.3389/fpsyg.2017.00076

**Published:** 2017-01-27

**Authors:** Yun Zhuang, Wenfeng Feng, Yu Liao

**Affiliations:** Department of Psychology, School of Education, Soochow UniversitySuzhou, China

**Keywords:** feedback-based learning, motivation, feedback valence, development, adolescents

## Abstract

The primary goal of the present study was to investigate how positive and negative feedback may differently facilitate learning throughout development. In addition, the role of motivation as a modulating factor was examined. Participants (children, adolescents, and adults) completed two forms of the guess and application task (GAT). Feedback from the Cool-GAT task has low motivational salience because there are no consequences, while feedback from the Hot-GAT task has high motivational salience as it pertains to receiving a reward. The results indicated that negative feedback leads to a reduction in learning compared to positive feedback. The effect of negative feedback was greater in adolescent participants compared to children and adults in the Hot-GAT task, suggesting an interaction between age and motivation level on learning. Further analysis indicated that greater risk was associated with a greater reduction in learning from negative feedback and again, the reduction was greatest in adolescents. In summary, the current study supports the idea that learning from positive feedback and negative feedback differs throughout development. In a rule-based learning task, when associative learning is primarily in practice, participants learned less from negative feedback. This reduction is amplified during adolescence when task-elicited motivation is high.

## Introduction

From the general development of physical and mental abilities to education in school, feedback-based learning is a key factor to successful learning beginning in infancy. Feedback-based learning refers to the ability to use performance feedback in the adjustment of subsequent behavior (Goldstein et al., 2003; Hounsell, 2003; Perera et al., 2008). Positive feedback signals that a previous response was correct and thus should be repeated in the future, while negative feedback signals that the past behavior was incorrect and as such, the behavior needs to be adjusted to successfully adapt to future similar situations.

Prior studies have explored aged differences in sensitivity to feedback-based learning. Interestingly, these studies have indicated that children tend to learn better from positive feedback. Adults, on the other hand, benefit more from negative feedback while adolescents show learning characteristics of both children and adults (Crone et al., 2004, 2008; Huizinga et al., 2006; van Duijvenvoorde et al., 2008; Peters et al., 2014). The changing sensitivity to positive and negative feedback with development may due to maturity level and engagement of various brain areas related to cognitive control.

The core brain areas of cognitive control, such as the prefrontal cortex (PFC) and the dorsal anterior cingulate cortex (dACC) undergo a prolonged developmental period (Fuster, 2002; Geier, 2013). These frontoparietal areas also exhibit increased task-related activity with age (Ordaz et al., 2013). Some studies suggest that there may be a quantitative shift in the recruitment of frontoparietal regions from positive feedback to negative feedback during development (van Duijvenvoorde et al., 2008; van den Bos et al., 2009; Peters et al., 2014). In sum, most prior studies have supported the theory of a shift from a “positive-learner” to a “negative-learner” from childhood to adulthood.

However, other studies using a probabilistic learning paradigm have suggested that children between 9 and 11 years old are more sensitive to negative feedback than positive feedback (Hämmerer et al., 2010). In addition, another study using a computational model to estimate the learning rate of children, adolescents, and adults, found that learning rate based on negative feedback decreased with age while learning rate based on positive feedback did not change with age (van den Bos et al., 2012). The age difference in learning rate is associated with an age-related increase in functional connectivity between the ventral striatum and the medial PFC, a pathway that shows increased activation during feedback processing (Münte et al., 2008; Camara et al., 2009). These data indicate that the changing sensitivity to positive and negative feedback during development is not related to differences in neural representation of learning signals *per se*, but rather in how learning signals are used to guide behavior and expectations (van den Bos et al., 2012).

The disparity in the above research may be due to the specific tasks applied by each study. The response to a rule-based associative learning task (van Duijvenvoorde et al., 2008; Peters et al., 2014) entirely depends on understanding the rules of the task. In rule-based learning, retrospective experience is used and is often implemented in a relatively “cool” context (i.e., card sorting). Feedback informs the learner whether a response was correct or incorrect. Unlike rule-based learning, the response to a probabilistic learning task (Hämmerer et al., 2010; van den Bos et al., 2012) depends on an estimate of an optimal option because the reward is only probabilistic. This type of learning relies not only on retrospective experience but also on prospective calculations of upcoming signals. Probabilistic learning is often implemented in a relatively “hot” context (i.e., gambling) where feedback comes in the form of a reward or punishment. It is possible that different types of learning recruit different brain regions that vary in their sensitivities to positive and negative feedback.

In addition to the different types of learning explored in previous studies, the role of motivation in learning varies among studies. Participants in a gambling game may be more motivated than participants performing a card-sorting task. In fact, prior studies have shown that high reward with high motivation increases performance. However, there is a point at which a high reward becomes detrimental to performance. This phenomenon is referred to as “the choking effect” (Chib et al., 2012, 2014). The choking effect is also apparent when losing a reward is at stake (Chib et al., 2014). It has been suggested that dopamine mediates the choking effect. More specifically, it is theorized that the choking effect brought on by over-motivation, due to an imbalance between activation in subcortical (reward-related) versus prefrontal (cognitive) regions. This imbalance leads toward a shift from more goal-directed behavior to the habitual guidance of behavior (Aarts et al., 2014). In sum, motivational level may also play an important role in the inconsistency between results of previous reports. Interestingly, adolescents are in a stage of imbalanced development of reward circuits and cognitive control circuits (Hagmann et al., 2010; Mills et al., 2014; Baker et al., 2015). The adolescent brain is much more sensitive to reward than that of a child or an adult, and as such, adolescents prefer risky choices (Somerville et al., 2010; Cassotti et al., 2011; Casey, 2015). In addition, adolescents tend to overvalue future reward while ignoring negative/neutral stimuli (Palminteri et al., 2016). It is therefore necessary to take motivational level into consideration to fully understand the development of feedback-based learning, especially during the adolescent period.

The current study was conducted to gain more clarity on how learning is differently modulated by feedback valence and motivation during development. Rule-based learning under different levels of task-elicited motivation was studied. Motivational levels were manipulated by modifying “cool” and “hot” executive function tasks into homogeneous rule-based learning tasks. Hot executive function tasks are considered more emotional and motivational than cool executive function tasks (Prencipe et al., 2011). The gambling task is a typical hot executive function paradigm (Kerr and Zelazo, 2004). Motivation was also directly manipulated in the “hot” task by applying a different magnitude of reward/punishment trial by trial. This design permits a direct comparison of performance under different motivational levels within one task context.

## Materials and Methods

### Participants

Ninety-nine healthy participants aged from 6 to 24 years old were initially enrolled in the study. Because the current study aimed to examine possible interactive effects between feedback valence and risk-taking behavior (e.g., betting on a risky rather than a conservative card), participants who were biased to choose only one option (risky or conservative) were excluded from the final analysis. The final sample included 65 participants who each completed at least eight trials of four possible combinations: risky choice with positive feedback; risky choice with negative feedback; conservative choice with positive feedback; conservative choice with negative feedback. There were 19 adults (nine females; *M* = 19.79 years, *SD* = 1.93, range = 18–24 years), 23 early adolescents (nine females; *M* = 151.57 months, *SD* = 16.81, range = 134–185 months), and 23 children (12 female; *M* = 107.83 months, *SD* = 14.90, range = 81–129 months).

All participants were recruited through local advertisements or referred by former volunteers. All participants reported normal or corrected-to-normal vision and an absence of neurological or psychiatric impairments. Prior to participation, informed consent was given by the guardian (children and adolescents) or by the participant (adults). Participants received rewards according to their performance on the tasks, and additional gifts were given to children and adolescents at the end of the session. All procedures for the current research were approved by the ethical committee of Soochow University.

### IQ Test for Children and Adolescents

IQ scores of child and adolescent participants were estimated using two subtests (Similarities and Block Design) of the Chinese Wechsler Intelligence Scale for Children (C-WISC). These subtests are considered to reliably represent the entire C-WISC test (Gong and Cai, 1993). Estimated IQ scores of children ranged from 92.29 to 131.52 (*M* = 114.99, *SD* = 11.29) while those of adolescents ranged from 89.15 to 126.03 (*M* = 109.46, *SD* = 10.41). Age differences were not significant. A standard intelligence test was not given to adults because they were all undergraduates or postgraduates of Soochow University and as such considered intellectually homogeneous.

### The Guess and Application Tasks (GAT)

Two versions of the guess and application task (GAT) were used in the current study: a cool executive function based task (referred to as Cool-GAT through the remainder of the text), and a hot executive function based task (referred to as Hot-GAT for the remainder of the text). The Cool-GAT is adopted from the rule selection and application task (Zanolie et al., 2008). The Hot-GAT is a matched version of Cool-GAT, designed by the current study. Detailed differences between the two tasks will be elaborated in the following description.

**FIGURE 1:**
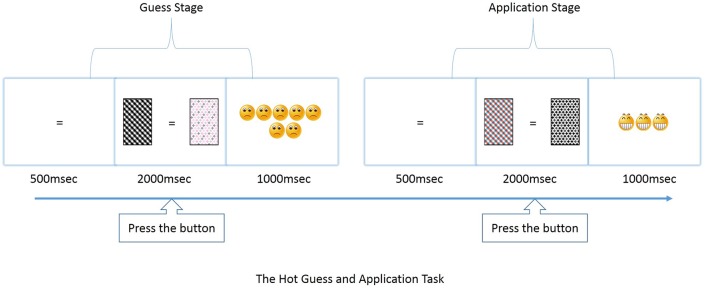
**Illustration of game paradigm and event time line for Hot guess and application task (GAT)**.

**Figure 1** illustrates an example of a trial of the Hot-GAT task. Each trial consisted of two stages: a *guessing stage* and an *application stage*. During *the guessing stage*, participants were asked to guess which of two cards would win in a given trial. A card with a black/white pattern and a card with a colored pattern were presented on each side of a screen with the position randomly assigned in each trial (50% of colored cards presented on the right side) to avoid position bias. Before the cards were displayed, a black or a colored “=” was presented in the center of the screen for 500 ms, indicating a greater risk was associated with the black/white card or the colored card, respectively. Positive or negative feedback was given immediately after participants made a choice by pressing the “A” or “L” button within 2 s after the cards were presented. Participants can press “A” button to choose the left card or the “L” button for the right card. Positive feedback (win) was presented with happy faces while negative feedback (loss) was presented with sad faces. The feedback included 5–7 faces following a risky choice and 1–3 faces following a conservative choice. The feedback revealed whether a black/white patterned or a color-patterned card was the winner in that particular trial. During the *application stage*, participants could apply the learned rule to make a choice between two new available cards with the same color features as was presented during the *guessing stage*. Again, the choice was made within 2 s and again, feedback with happy/sad faces followed, lasting for 1 s.

Accuracy and reaction time (RT) were both recorded for the *guessing* and *application stages*. To increase motivation, participants were informed that they would gain/lose one point for each happy/sad face, respectively and the final number of points determine the amount of treats (candy) they would get. For control, the pattern (not the color) of the colored card and black/white card was interchangeable within each trial to create new exemplars in *the application stage*. A black screen was presented for 1–1.5 s to provide salient information that a trial had ended.

To ensure that the rules of the game were well understood, thorough instructions were given immediately prior to each test. Key instruction points for the rules of the Hot-GAT task included: (1) they would bet on a risky or a conservative card in *the guess stage* and receive feedback showing a win (smiling faces) or a loss (sad faces); (2) the color of the cue symbol (“=”) indicated which card was risky in the current trial, the pattern on the card was irrelevant; (3) participants could learn which card would win based on feedback they receive during *the guessing stage*; (4) in order to win points, participants should apply knowledge gained during the *guessing stage* during *the application stage*; (5) one smiling face earns +1 point while one sad face earns -1 point, and the more points gained, the greater the candy reward. The Cool-GAT task shared the same overall “guess and apply” structure and the same timeline as the Hot-GAT game. However, instead of taking risks to guess which card would win, participants were asked to guess which color/shape would win by selecting one of two presented cards during *the guessing stage*. A cue presented in the center of the screen informed the participant on whether the current trial was a color game (colored “=”) or a shape game (black “=”). Essentially, the Cool-GAT game required the participant to learn which dimension is relevant in each trial; color or shape. The Hot-GAT game, on the other hand, required the participant to learn which color is associated with a higher payoff in each trial. In addition, instead of using emotional faces, feedback in Cool-GAT games was given with a“√” (positive feedback) or a “×” (negative feedback) in the center of the screen. The critical difference is that the feedback stimuli for the Cool-GAT game did not carry any direct emotional information while the feedback stimuli for the Hot-GAT game was associated with emotional faces. As a control for the Cool-GAT game, the relevant dimension (color/shape) within each trial was fixed, while the irrelevant dimension switched within each pair of cards.

All participants finished 60 trials of the Hot-GAT game and 60 trials of the Cool-GAT game. Each set of trials was divided into three blocks of 20 trials each. To encourage participants, the total score of each *application stage* trial was shown in the center of the screen at the end of each block. Prior to test trials participants were given an explanation of the rules and practiced four five trials for each of the games without time limit. The sequence of the two games (Hot-GAT and cool-GAT) was counterbalanced.

During the *guessing stage*, trials with positive or negative feedback were randomly delivered at a 5/5 ratio. However, because the feedback that each participant received in *the guessing stage* was partially choice dependent, the final amount of positive or negative feedback each participant experienced was slightly different. In the Cool-GAT games, participants completed 28.8 ± 3.5 trials with positive feedback and 29.9 ± 3.5 trials with negative feedback during *the guessing stage*. Similarly, in the Hot-GAT games, participants completed 28.8 ± 3.0 trials with positive feedback and 29.9 ± 3.0 trials with negative feedback during *the guessing stage*.

### Analysis

The primary goal of the present study was to advance our understanding of how positive and negative feedback may differently facilitate learning throughout development and to examine the modulatory role of motivation.

A three factor (feedback valence × age group × task) repeated-measures ANOVA was performed on accuracy and RT data. Age group was treated as a between-subject variable while feedback valence and task were treated as within-subject variables. To reveal a possible separate modulation of positive and negative feedback for different tasks, a feedback valence × age group repeated-measures ANOVA was also performed on the accuracy and RT data for the Cool-GAT games and the Hot-GAT games separately. In addition, because the Hot-GAT game included two levels of risk taking, a feedback valence × age group × risk taking repeated-measures ANOVA was applied to explore possible modulation by task-elicited motivation on learning efficiency. *Post hoc* tests were applied when interaction effects were significant.

Learning from negative feedback in a rule-based associative learning task requires behavior adjustment and is therefore more difficult than learning from positive feedback. Thus, a general reduction in accuracy and speed of response after negative feedback was predicted. This reduction in accuracy and speed should be relatively stable across age groups if participant sensitivity to positive and negative feedback develop synchronously over time. However, it is possible that a certain age group is more sensitive to one type of feedback over the other. Thus, a one-way ANOVA was performed to examine the reduction of accuracy after negative feedback compared to positive feedback across the three age groups. In addition, for the Hot-GAT games, a two-way repeated-measures ANOVA was applied to examine developmental changes in sensitivity to negative and positive feedback under varying risk-taking levels.

The correlation between IQ and task performance, including accuracy and RTs across all conditions (the Cool-GAT game, the Hot-GAT game, positive and negative feedback, age groups, and risk level) was calculated after controlling for age and gender. To examine possible gender effects, A mixed-measures ANOVA of gender × feedback valence × task × age group was performed on the accuracy and RTs of both tasks, and a mixed-measures ANOVA of gender × feedback valence × risk taking × age group on the accuracy and RTs of the Hot-GAT.

## Results

Participants in all age groups displayed learning in both the Cool-GAT and the Hot-GAT tasks. Accuracy dramatically increased during *the application stage* compared to the *guessing stage* in both GAT tasks. On average, for the Cool-GAT task, accuracy level increased 25.64 ± 17.51% for children, 27.87 ± 14.11% for adolescents and 43.52 ± 10.14% for adults. For the Hot-GAT task, accuracy level increased 30.48 ± 16.90% for children, 33.26 ± 17.36% for adolescents and 45.64 ± 7.35% for adults. The increased accuracy level indicates that learning occurred during *the guessing stage* in all age groups. Thus, only accuracy and RT from *the application stage* was analyzed since these measures reflect the learning effect of the previous stage. Overall, as shown in **Table 1**, participants in all age groups performed well (all greater than 70% accuracy) with increasing accuracy with age. Age differences are elaborated in the next section.

**Table 1:** Performance on the Cool-GAT task and the Hot-GAT task.

Age group (years of age)	Task	Cool-GAT (*M* ±*SD*)		Hot-GAT (*M* ±*SD*)	
	Feedback	Positive	Negative	Positive	Negative
Children (6–10)	ACC (%)	76.4 ± 21.0	70.3 ± 16.5	80.8 ± 17.3	78.3 ± 17.4
*n* = 23	RT (ms)	736 ± 203	803 ± 226	723 ± 170	768 ± 186
Adolescents (11–16)	ACC (%)	82.7 ± 14.6	74.6 ± 13.6	87.9 ± 13.7	76.4 ± 20.6
*n* = 23	RT (ms)	692 ± 127	777 ± 180	756 ± 193	796 ± 193
Adults (18–25)	ACC (%)	94.4 ± 12.0	89.7 ± 11.1	97.4 ± 5.4	93.1 ± 6.4
*n* = 19	RT (ms)	537 ± 163	624 ± 187	587 ± 122	655 ± 164

### The Differential Effects of Positive and Negative Feedback on Learning among Age Groups

**FIGURE 2:**
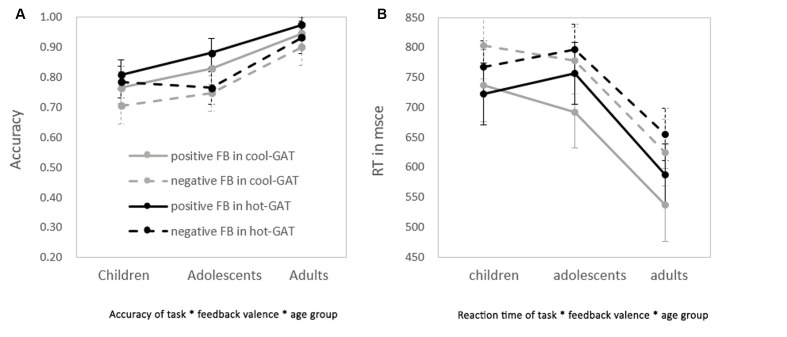
**Performance on the application stage of the Cool-GAT and the Hot-GAT after receiving positive/negative feedback in the guessing stage. (A)** The accuracies of each condition in each age group. **(B)** The reaction time (RT) of each condition in each age group. Dashed lines represent the performance of trials with negative feedback; solid lines represent trials with positive feedback. Darker lines represent trials from the hot-GAT game; lighter lines represent trials from the cool-GAT game.

In general, as shown in **Figure 2A**, accuracy was greater in the Hot-GAT task than in the Cool-GAT task [*F*(1,62) = 5.18, *p* < 0.05, η^2^ = 0.08]. In addition, positive feedback led to greater accuracy than negative feedback [*F*(1,62) = 44.75, *p* < 0.01; η^2^ = 0.42]. There was a significant effect of age on accuracy [*F*(2,62) = 11.64, *p* < 0.01, η^2^ = 0.27]. An LSD *post hoc* test showed that adults performed significantly better than adolescents and children (*ps* < 0.05). However, accuracy did not significantly differ between children and adolescents.

In addition to the main effects, there was a significant interaction between age group and feedback valence; [*F*(2,62) = 4.02, *p* = 0.02, η^2^ = 0.12]. An LSD *post hoc* test revealed that adults performed significantly better than children and adolescents (*ps* < 0.05) after positive feedback, their accuracy however, did not differ across age group after negative feedback. There was a tendency for accuracy to be increased more after positive feedback (increased 6.7%) than negative feedback (increased 1.2%) in adolescents compared to children, but this effect was not statistically significant. No other significant interaction effects were found.

### The Differential Effects of Positive and Negative Feedback on Learning in Each Task

For the Cool-GAT task, performance based on positive feedback was significantly more accurate than performance based on negative feedback [*F*(1,62) = 17.05, *p* < 0.01, η^2^ = 0.22]. There was a significant effect of age on performance in the Cool-GAT task [*F*(2,62) = 9.53, *p* < 0.05, η^2^ = 0.24]. An LSD *post hoc* test showed that adults performed significantly better than adolescents (*p* < 0.01) and children (*p* < 0.01) while there was no significant difference between children and adolescents. To directly measure how the differences in learning from positive feedback and negative feedback may change with age, a one-way ANOVA was performed to examine accuracy differences between trials with positive feedback and negative feedback during *the guessing stage*. The result showed no main effect for age group (mean differences were 6.1 ± 2.6%; 8.2 ± 2.6%; 4.7 ± 2.8%; children, adolescents, and adults, respectively). There was no interaction between feedback and age groups.

Similar to the Cool-GAT task, performance following positive feedback was significantly more accurate than after negative feedback during the Hot-GAT task [*F*(1,62) = 22.32, *p* < 0.01, η^2^ = 0.27]. In addition, there was a significant age effect in the Hot-GAT task [*F*(2,62) = 7.22, *p* < 0.01, η^2^ = 0.19]. However, in contrast to the results for the Cool-GAT task, there was a significant interaction between feedback valence and age group in the Hot-GAT task [*F*(2,62) = 4.87, *p* = 0.01, η^2^ = 0.14]. *Post hoc* tests revealed that greater accuracy after positive feedback was only significant for adults and adolescents (*ps* < 0.01). Children, on the other hand performed equally well after receiving positive and negative feedback. Performance after positive feedback increased with age. Adults performed significantly better than children (*p* < 0.01) and adolescents (*p* < 0.03) after positive feedback while adolescents performed marginally better than children (*p* = 0.08). The increase in performance was significantly greater for adults after negative feedback compared to adolescents and children (*ps* < 0.01), while the increase in performance after negative feedback was not different between children and adolescents. To further investigate the different effects of positive and negative feedback on learning in the Hot-GAT games, differences in accuracy between positive and negative feedback were calculated for each age group. A one-way ANOVA showed a significant main effect for age group; [*F*(2,62) = 4.87, *p* = 0.01, η^2^ = 0.14]. An LSD *post hoc* test showed that the increase in accuracy for adolescents (11.5 ± 12.7%) was significantly larger than for children (2.5 ± 9.6%) and adults (4.2 ± 7.4%) (*p* < 0.01 and *p* < 0.03, children and adults, respectively). Children and adults did not significantly differ in accuracy between positive and negative feedback. In sum, these results suggest that overall, participants learned more from positive feedback than from negative feedback. Interestingly, the difference in performance after positive feedback and negative feedback varies with age. Adolescents were the most sensitive to feedback valence, as they showed the largest difference in learning between positive and negative feedback conditions.

There was no main effect of task on RT. However, as shown in **Figure 2B**, RT after negative feedback was significantly longer than after positive feedback [*F*(1,62) = 54.00, *p* < 0.01, η^2^ = 0.47]. In addition, there was an effect of age on RT [*F*(2,62) = 7.17, *p* < 0.01, η^2^ = 0.20]. A *post hoc* test showed that the RT of adults was significantly shorter than that of adolescents and children (*ps* < 0.01). Children and adolescents did not differ in RT. No other significant interactions were found.

### Interaction between Risk Level and Feedback Valence in the Hot-GAT Task

**FIGURE 3:**
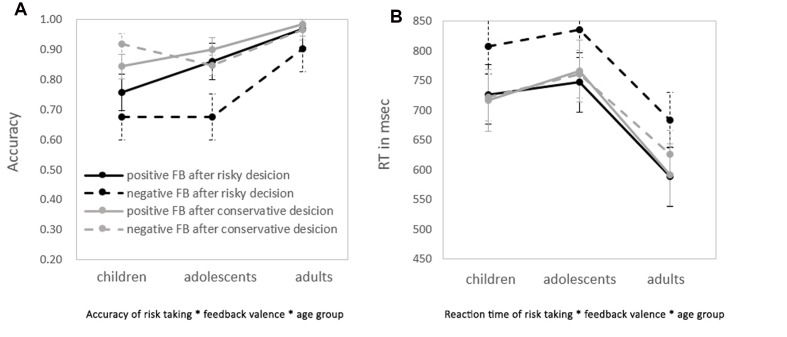
**Performance on the application stage of the Hot-GAT when risky/conservative choices followed by negative/positive feedback**. **(A)** The accuracies of each condition in each age group. **(B)** The RT of each condition in each age group. Dashed lines represent the performance of trials with negative feedback; solid lines represent trials with positive feedback. Darker lines represent trials with risky choices; lighter lines represent trials with conservative choices.

Accuracy and RT under the four conditions in the Hot-GAT task are shown in **Figure 3**. Consistent with the previous analysis, the repeated-measures ANOVA on the accuracy of feedback valence × risk taking × age group revealed a significant main effect of feedback valence; [*F*(1,62) = 19.42, *p* < 0.01, η^2^ = 0.24], with better performance after positive feedback than negative feedback. In addition, there was a significant effect of age on accuracy [*F*(2, 62) = 7.26, *p* < 0.01, η^2^ = 0.19]. The LSD *post hoc* test showed that adults performed significantly better than adolescents and children (*ps* ≤ 0.01). There was no statistically significant difference in accuracy between children and adolescents. Remarkably, there was a main effect of risk-taking [*F*(1,62) = 24.50, *p* < 0.01, η^2^ = 0.28]. Accuracy was significantly worse when participants made a risky choice (i.e., cards with large gain/loss) compared to a conservative choice (i.e., cards with small gain/loss) during the *guessing stage* (See **Table 2** for descriptive data). The three-way interaction was not statistically significant.

**Table 2:** Performance on the Hot-GAT task for different risk levels.

Age group (years of age)	Risk taking	Risky (*M* ±*SD*)		Conservative (*M* ±*SD*)	
	Feedback	Positive	Negative	Positive	Negative
Children (6–10)	ACC (%)	75.7 ± 24.8	67.5 ± 27.3	84.3 ± 20.8	91.7 ± 9.5
*n* = 23	RT (ms)	727 ± 174	807 ± 206	717 ± 184	722 ± 186
Adolescents (11–16)	ACC (%)	85.9 ± 19.9	67.4 ± 26.9	89.8 ± 13.7	84.6 ± 22.7
*n* = 23	RT (ms)	747 ± 171	836 ± 208	766 ± 223	761 ± 199
Adults (18–25)	ACC (%)	96.7 ± 7.0	90.2 ± 9.6	98.4 ± 4.0	96.5 ± 7.3
*n* = 19	RT (ms)	588 ± 124	683 ± 150	592 ± 156	627 ± 197

There was a significant interaction between feedback valence and risk taking [*F*(1,62) = 10.24, *p* < 0.01, η^2^ = 0.14]. A *post hoc* analysis revealed that when a risky choice was made, participants performed significantly better after positive feedback than after negative feedback (*p* < 0.01). However, participants performed equally well after positive and negative feedback when conservative choices were initially made. We further compared performance between the four possible combinations (risky choice with positive/negative feedback; conservative choice with positive/negative feedback) across age using paired *t*-tests. Adolescents and adults performed significantly worse when making a risky choice followed by negative feedback than in any of the three other conditions (all *ps* < 0.05). Children followed the same pattern but the difference between conditions was not statistically significant (*p* = 0.07). There were no significant differences between risky choices with positive feedback, conservative choices with positive feedback and conservative choices with negative feedback in performance. These results suggest that learning from negative feedback is more difficult, especially when risk is high.

To further explore reduced learning from negative feedback, differences in accuracy between positive and negative feedback following risky choices or conservative choices were calculated separately for each age group, by subtracting their accuracy followed negative feedback from their accuracy followed positive feedback for risky and conservative condition respectively. A repeated-measures ANOVA revealed main effects for both risk and age [*F*(1,62) = 10.24, *p* < 0.01, η^2^ = 0.14 and *F*(2,62) = 7.83, *p* < 0.01, η^2^ = 0.20; risk and age, respectively]. An LSD *post hoc* test showed that the difference in accuracy between positive and negative was significantly greater in adolescents (11.8 ± 2.1%) than children (0.4 ± 2.1%) (*p* < 0.05) and adults (4.2 ± 2.3%) (*p* < 0.02). There was no significant difference between children and adults. These results indicate that learning from positive and negative feedback is differentially modulated by motivation (risk level) for different age group. Learning from negative feedback is most difficult during adolescence when motivation is high. A repeated measures ANOVA for RT was performed. There was a main effect of risk [*F*(1,62) = 10.00, *p* < 0.01, η^2^ = 0.14]. RT was significantly longer for risky decisions than for conservative decisions at all age groups. There was a main effect of age on RT [*F*(2,62) = 4.77, *p* = 0.01, η^2^ = 0.13]. An LSD *post hoc* test showed that the RT of adults was significantly faster than adolescents and children (*ps* ≤ 0.05).There were no statistically significant differences between children and adolescents in RT. Lastly, there was a main effect of feedback valence [*F*(1,62) = 16.05, *p* < 0.01, η^2^ = 0.21], with faster RTs after positive feedback than negative feedback.

There was a significant interaction between risk and feedback valence on RT [*F*(1, 62) = 15.11, *p* < 0.01, η^2^ = 0.20]. An LSD *post hoc* test revealed that when risky choices were made, the RT after positive feedback was significantly shorter than that after negative feedback (*p* < 0.01). However, there was no difference between positive and negative feedback on RT when conservative choices were made. As shown in **Figure 3**, RT in the risky-negative feedback condition was significantly longer than all other conditions among all age groups (*ps* < 0.01). There were no significant differences in RT among the three other conditions. These results indicate that learning from negative feedback utilizes more cognitive resources resulting in longer RTs than learning from positive feedback. This effect is strengthened in cases where risky choices were made. No additional interactions effects were found regarding RT.

### Correlation between IQ and Task Performance

General accuracy on the Cool-GAT task was significantly correlated with IQ score after controlling for age, gender, and other variables (*p* < 0.01, *r* = 0.39). There were no other significant correlations between IQ and task performance.

### Gender Differences

There were no significant main effects of gender or interactions between gender and feedback valence, task or age group on accuracy or RT.

## Discussion

To investigate the modulatory effect of motivation on feedback-based learning across different developmental stages, we examined child, adolescent and adult participant performance on two rule-based learning tasks: a Cool-GAT task and a Hot-GAT task. In both GAT tasks, the participants first guessed which one out of two cards would “win.” The participants were then immediately given positive or negative feedback. Learning was measured by how well participants performed in the *application stage* where the same rules were applied.

The results of the Cool-GAT game were consistent with previous studies using the same task with similarly aged participants (van Duijvenvoorde et al., 2008). In general, adults performed better than the younger groups. Children, adolescents, and adults all performed more accurately and responded faster after receiving a positive feedback than after receiving a negative feedback in the *application stage*. Comparatively, a behavioral adjustment (i.e., prediction was incorrect) is required after negative feedback, causing a reduction in successful learning and speed of response. This reduction, however, showed different developmental trends in the current study. According to van Duijvenvoorde et al. (2008), the difference in accuracy between positive and negative feedback decreased with age, suggesting that children are more sensitive to feedback valence. In the current study, however, the reduction in learning from negative feedback compared to positive feedback did differ significantly with age. In fact, the present results found that the difference is slightly larger for adolescents (8.2 ± 2.6%) than children (6.1 ± 2.6%) or adults (4.7 ± 2.8%). The difference in results may be due to better overall performance of the children in the current sample, given that accuracy based on negative feedback (70%) was almost the same as the overall performance of both positive and negative feedback in the study by van Duijvenvoorde et al. (2008). The superior performance by the children in the current study may be explained by earlier maturation of executive functioning for Chinese children. Several studies have demonstrated that children who are raised in China or are exposed to Chinese culture perform significantly better on measures of cool executive function (Lewis et al., 2006; Sabbagh et al., 2006) and hot executive function (Qu et al., 2012) compared to age-matched Western children. Consistently, it has been reported that the 7-repeat allele of the dopamine receptor gene (DRD4) which is linked with poor executive function (Faraone et al., 2001), is relatively rare in Asian people compared with North American people (Chang et al., 1996). As some research suggests, following the traditional Confucian philosophy of obedience and impulse control are emphasized in the Chinese education system (Chen et al., 1998). Thus, self-control and regulation of behavior is encouraged in Chinese children.

The overall trends in learning for the Hot-GAT game were similar to those for the Cool-GAT game, except in adolescents. Overall, participants showed a reduction in learning from negative feedback compared to positive feedback. Interestingly, these reductions were amplified in adolescents. In other words, the reduction in learning after negative feedback is significantly greater for adolescents than for children or adults in the Hot-GAT game. As mentioned, the general reduction in learning from negative feedback may result from the need to adjust behavior because a correction of an initial response is required. The changes in reduction in learning in response to negative feedback at different ages suggests an asynchrony in the development of sensitivity to feedback valence. The amplified reduction in learning from negative feedback in the Hot-GAT game for adolescents suggests that sensitivity to negative feedback may be modulated by motivational state.

It may be argued that the amplified reduction in learning from negative feedback in adolescents in the Hot-GAT game is caused by overall task differences, not necessary by the high motivation level elicited by the game. However, during the *guessing stage*, when participants chose a riskier option by selecting a higher gain/loss card, learning was reduced to a greater extent after negative feedback compared to positive feedback in all age groups. Again, the reduction in learning was greatest for adolescents compared to children or adults. Surprisingly, there were no differences in learning observed from positive compared to negative feedback in the conservative condition (participants selected a low gain/loss card during *the guessing stage*) where the motivation elicited by the task is considerably lower. These data strongly indicate that learning from negative feedback is modulated by reward-induced motivation level. Participants learned less when motivation was high. This detrimental effect of “over-motivation” is greatest during adolescence.

Compared to other developmental periods, adolescents process reward differently. Behavioral evidence demonstrates that scores on sensation-seeking, risk preference, and reward sensitivity increase from age 10 until mid-adolescence, peaking between 13 and 16 years of age, and then decline (Steinberg et al., 2008; Ernst, 2014). These data are consistent with the adolescent sample in the current study. In addition, brain imaging data consistently show that the nucleus accumbens, a brain region that is associated with risky choices, exhibits exaggerated activity to outcomes in adolescents compared to children and adults (Ernst et al., 2005; Galvan et al., 2006). The heightened sensitivity may alter adolescent cognitive processing associated with risky choices. Consistent with this inference, previous studies have demonstrated that adolescents report higher emotional arousal than adults when receiving rewards (Ernst et al., 2005) and experience slower heart rate upon receipt of negative feedback compared to positive feedback (van Duijvenvoorde et al., 2013). In addition, over-motivation may be detrimental to task performance as demonstrated by the “choking effect” (Chib et al., 2012, 2014). Specifically, high reward-induced motivation affects cognitive performance on a bell-shaped curve, in that very high reward can decrease performance. This over-motivation effect may be due to an imbalanced activation between reward processing regions located in the subcortical area and the cognitive control center from PFC (Aarts et al., 2014). Specifically, for people with a high baseline capacity for dopamine synthesis, high-level motivation might “overdose” the dopaminergic system in the reward circuits, thereby impairing rather than improving cognitive control (Cools and D’Esposito, 2011). Based on these previous studies, it is reasonable to infer that adolescent, right in the period that reward circuits and cognitive control circuits show imbalanced maturation, might be more sensitive to motivational change during behavioral adjustments. In another word, it is more plausible that motivation could impair adolescents’ cognitive control. As a result, learning from negative feedback become harder for a correction is needed in such circumstances.

It is possible that the amplified reduction in learning as a result of negative feedback during adolescence may be limited to associative learning. As mentioned earlier, associative learning, such as the GAT tasks, is usually rule-based, and relies on a trial-and-error experience. Once the rule is learned, however, the correct response is obvious. Adaptive learning is another frequently used feedback-based learning task. Adaptive learning is probability based and relies on both experience and prediction of future events. The prediction in this case is based on probability, and thus the answer is always uncertain. It is possible that different types of learning recruit different strategies or brain areas. Future brain imaging studies are necessary to further explore the possibility of the involvement of different brain circuits in these two different types of learning tasks.

Despite positive findings, there are limitations to the current study. The aim of the present study is to compare the learning effects after risky and conservative choice that followed by positive and negative feedback. A fair amount of trials is necessary to investigate the learning effect under each condition. Therefore, participants who extremely prefer to take risks or avoid taking risks were excluded as outliers from the final analysis. Thus, the current results do not represent this population. On the other hand, this within-subject comparison makes the results more reliable for highlighting the modulation of motivation on feedback-based learning from positive and negative feedback. However, it is worth to note that individuals with differed risk preference may differently react to the same positive or negative feedback. Further studies are necessary to explore the effect of these individual differences in feedback-based learning.

In summary, the current study supports the idea that learning from positive feedback and negative feedback develops separately. In a rule-based learning task, when associative learning is primarily in practice, negative feedback was detrimental to the learning process as compared to positive feedback. This reduction in learning as a consequence of negative feedback is amplified during adolescence when task-elicited motivation is high.

## Ethics Statement

The study was supported by Ethics Committee of Soochow University (ECSU). Firstly, the experimenter explained to participants or their guardians and promised that this study respects the dignity and worth of all people, and the rights of individuals to privacy, confidentiality, and self-determination. Participants and their guardians were informed that your participation in this research is entirely voluntary, and this experiment is totally harmless. Secondly, the experimenter explained what participants have to do in this research. When the participant and their guardians agreed to participant in the research, they were asked to sign the informed consent. The guardian of each minor participant gave written informed consent.

## Author Contributions

YZ contributed to experimental design, data collection, data analysis, and paper writing. WF contributed to experimental design and paper writing. YL contributed to experimental design, data analysis, and paper writing.

## Conflict of Interest Statement

The authors declare that the research was conducted in the absence of any commercial or financial relationships that could be construed as a potential conflict of interest.
